# Supplementary material for: Defining diurnal fluctuations in mouse choroid plexus and CSF at high molecular, spatial, and temporal resolution
Source: Nat Commun. 2023 Jun 22;14:3720. doi: 10.1038/s41467-023-39326-3 (PMC10287727; doi:10.1038/s41467-023-39326-3)
Supplement: Supplementary file 1 — Supplementary Information [file 41467_2023_39326_MOESM1_ESM.pdf]

Supplementary Figures 1-6 and legends.

Fame, et al. 2023

Supplementary Figure 1. The ChP exhibits rhythmic expression of core clock components in phase with liver.

Supplementary Figure 2. The ChP differentially translates secreted proteins during the light vs. dark phases.

Supplementary Figure 3. Ttr is preferentially translated in ChP during the dark phase and CSF metabolites differ between the light and dark phases.

Supplementary Figure 4. Fiber photometry in *Ttr*^{mNeonGreen} mice reveals TTR cycles over multiple days that are light independent.

Supplementary Figure 5. *In vivo* ChP metabolic components and metabolites are diurnally regulated and *ex vivo* ChP metabolism is normalized between light and dark phases.

Supplementary Figure 6. ChP barrier components and permeability are diurnally regulated.

Supplementary Figure 1

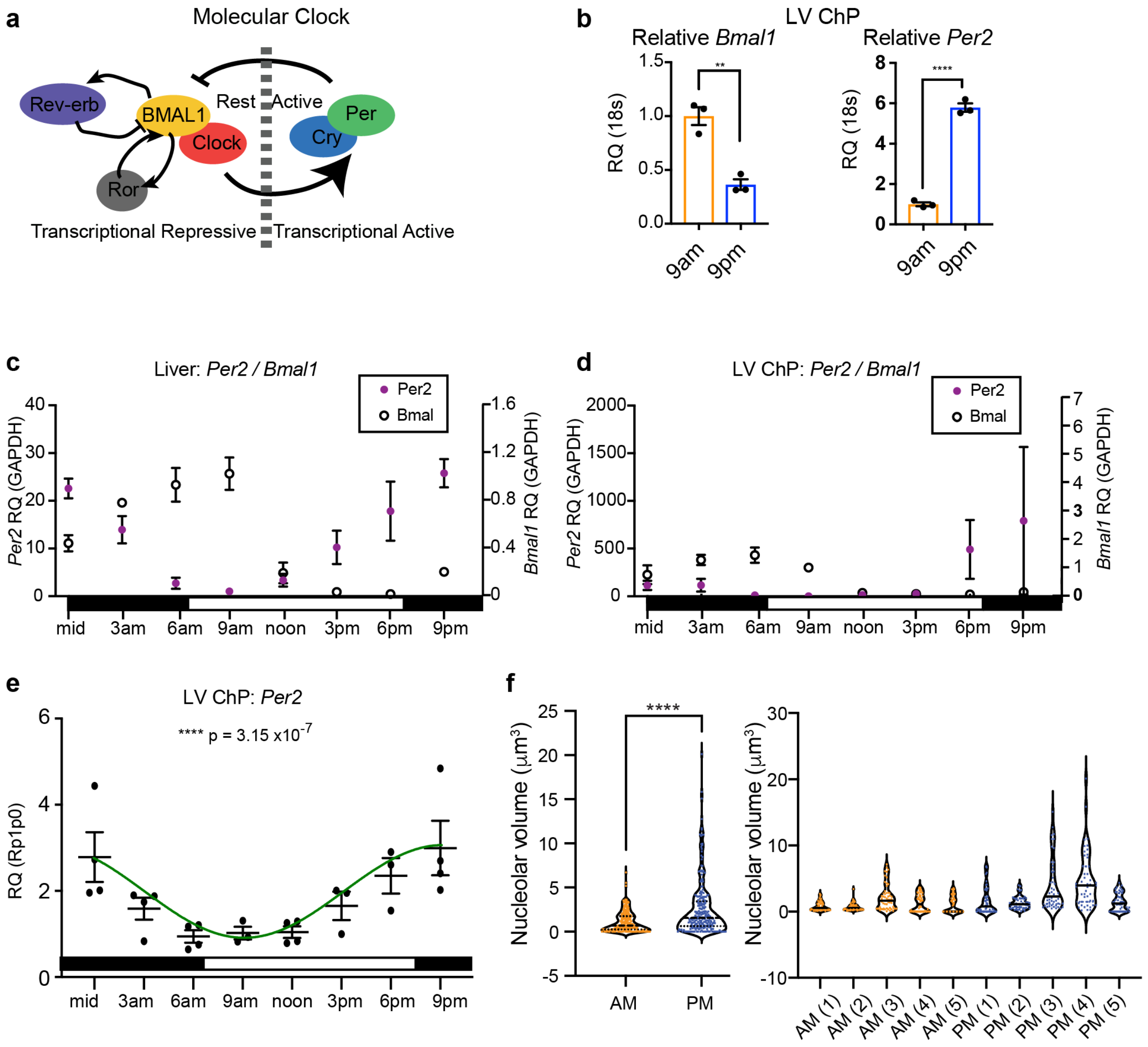

Supplementary Figure 1. The ChP exhibits rhythmic expression of core clock components in phase with liver. (**a**) Schematic of the major components of the molecular circadian clock. (**b**) RT-qPCR of *Bmal1*and *Per2* in LV ChP at 9 a.m. and 9 p.m. N = 3 biologically independent animals per time. Student's unpaired two-tailed t-test. Data are presented as mean values ± standard deviation (SD). (**c**) RT-qPCR of *Bmal1* and *Per2* in livers from the same mice analyzed in (D) across a whole day at 3-hour intervals showed the well-known anti-correlated rhythms. Data are presented as mean values ± standard deviation (SD). (**d**) RT-qPCR of *Bmal1* and *Per2* in LV ChP from the same mice analyzed in (c) across a whole day at 3-hour intervals showed anti-correlated rhythms. Data are presented as mean values ± standard deviation (SD). (**e**) RT-qPCR of *Per2* normalized to rplp0 across a whole day at 3-hour intervals demonstrates significant rhythmicity over the 24-hour day ($p = 3.15x10^{-7}$). N = 3 biologically independent animals per time. Statistical values generated by RAIN analysis. Data are presented as mean values ± standard deviation (SD). (**f**) Quantification of nucleolar volume plotting each nucleolus by time of day ($p < 0.0001$) and plotting each nucleolus grouped by individual animal. N = 5 biologically independent animals per time in 2 independent experiments. Two-tailed unpaired Welch's t-test. Data are presented as violin plot with solid bar denoting the median and broken lines representing quartiles. Male mice were analyzed. Source data are provided as a Source Data file (Source_Data).

Supplementary Figure 2

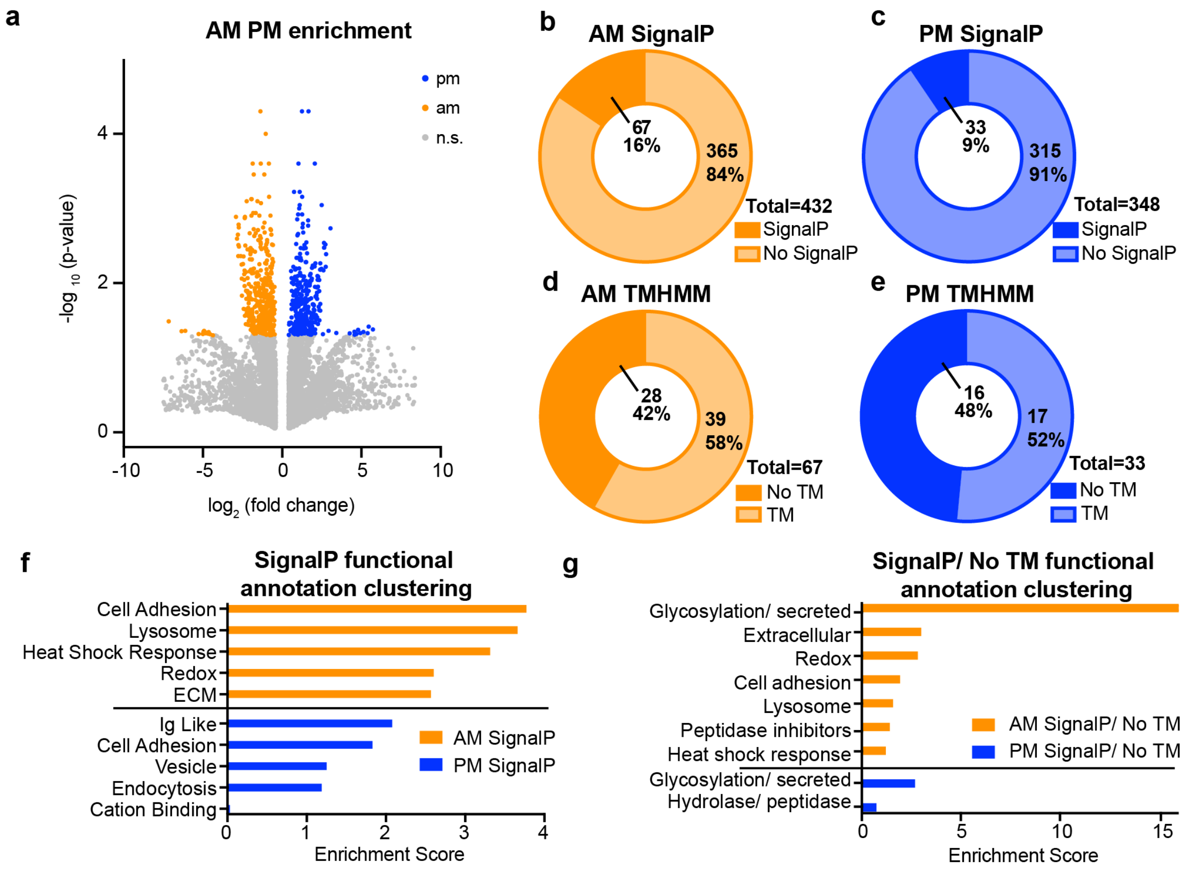

Supplementary Figure 2. The ChP differentially translates secreted proteins during the light vs. dark phases. (**a**) Volcano plot of those transcripts that are associated with the L10a-GFP ribosomal subunit that are enriched at 9 a.m. (orange) and 9 p.m. (blue) ChP epithelial cells compared to those that are not significant (n.s.; grey). (**b-c**) Proportion of enriched genes at 9 a.m. (orange) and 9 p.m. (blue) ChP with predicted signal peptides using SignalP. (**d-e**) Proportion of enriched genes at 9 a.m. (orange) and 9 p.m. (blue) ChP with predicted transmembrane domains using TMHMM. (**f**) Functional annotation clustering for those enriched genes at 9 a.m. (orange) and 9 p.m. (blue) ChP with predicted signal peptides using SignalP. (**g**) Functional annotation clustering for those enriched genes at 9 a.m. (orange) and 9 p.m. (blue) ChP with predicted transmembrane domains using TMHMM. Male mice were analyzed. Source data are provided as a Source Data file (Source_Data).

Supplementary Figure 3

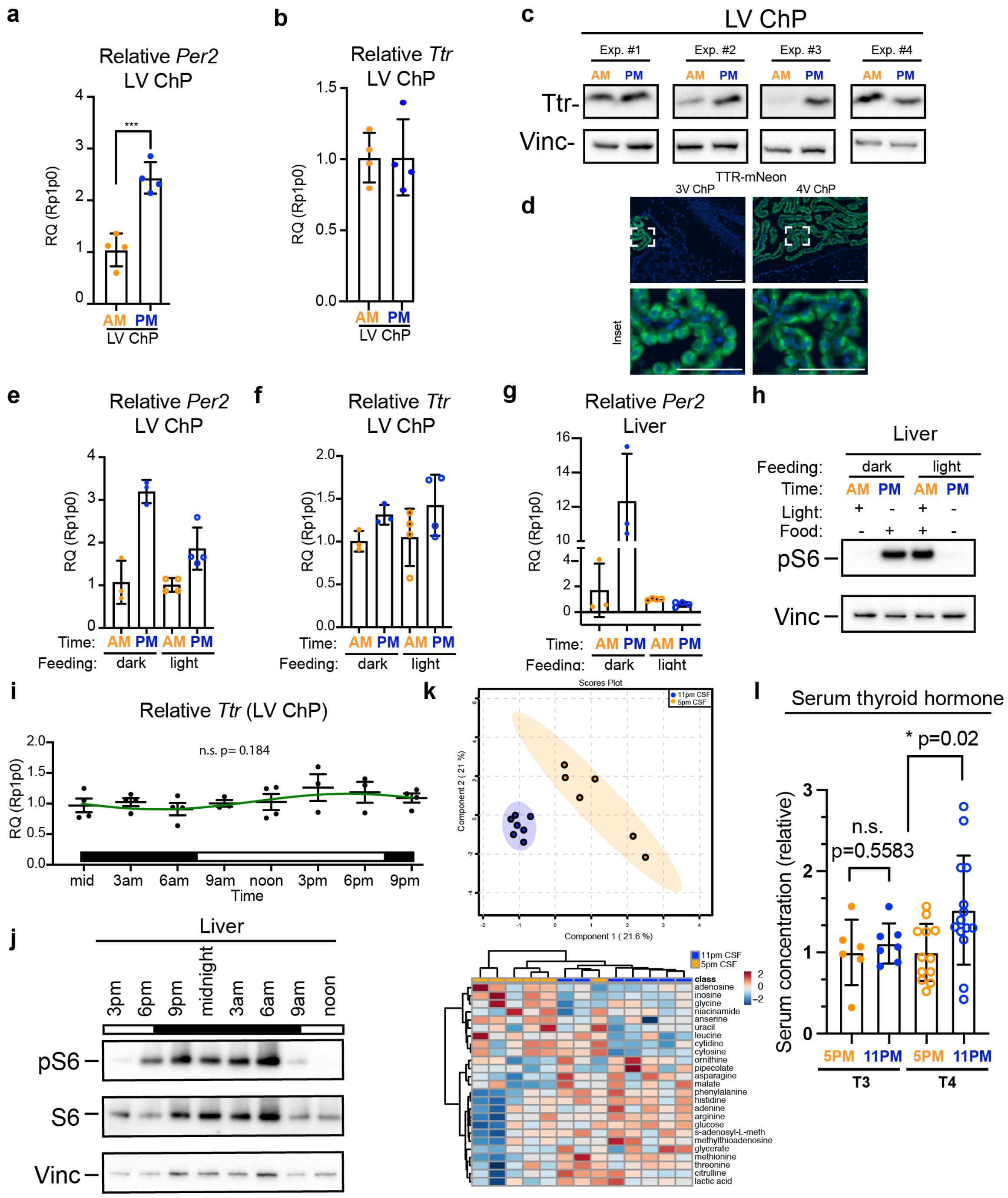

Supplementary Figure 3. *Ttr* is preferentially translated in ChP during the dark phase and CSF metabolites differ between the light and dark phases. (**a**) RT-qPCR of *Per2* in LV ChP at 9 a.m. and 9 p.m. N = 4 biologically independent animals per time. Student's unpaired two-tailed t-test. Data are presented as mean values ± standard deviation (SD). (**b**) RT-qPCR of *Ttr* in LV ChP at 9 a.m. and 9 p.m. N = 4 biologically independent animals per time. Student's unpaired two-tailed t-test. Data are presented as mean values ± standard deviation (SD). (**c**) Immunoblotting of LV ChP protein extracts for TTR. N = 4 biologically independent animals per time. (**d**) Fluorescence images of ChP from $Ttr^{mNeonGreen}$ mice in 3V and 4V ChP. Scale bar = 100 μm; inset scale bar = 50 μm. (**e**) RT-qPCR for *Per2* in LV ChP showed that the intrinsic clock was not affected by feeding. N = 4 biologically independent animals per time. Data are presented as mean values ± standard deviation (SD). (**f**) RT-qPCR for *Ttr* in LV ChP showed that *Ttr* transcript was not affected by feeding. N = 4 biologically independent animals per time. Data are presented as mean values ± standard deviation (SD). (**g**) RT-qPCR for *Per2* in liver showed that the liver clock was affected by feeding. N = 4 biologically independent animals per time. Student's unpaired two-tailed t-test. Data are presented as mean values ± standard deviation (SD). (**h**) Immunoblotting of liver protein extracts for p-S6 showed that increased phosphorylation levels at 9 p.m. is dependent on feeding. (**i**) RT-qPCR for *Ttr* in LV ChP every 3 hours demonstrates lack of significant rhythmicity over the 24-hour day ($p = 0.184$) from RAIN analysis. N = 4 biologically independent animals per time. (**j**) Immunoblotting of p-S6 in liver across a whole day at 3-hour intervals showed sharp upregulation of p-S6 during the dark phase. N = 4 biologically independent animals per time. (**k**) Metabolomics clustering reveals district metabolite profile from CSF at 5 p.m. (orange) and 11 p.m. (blue). N = 6 biologically independent animals at 9 a.m. and N = 7 biologically independent animals at 9 p.m. (**l**) Serum thyroid hormone levels and 5 p.m. (orange) and 11 p.m. (blue) showed difference in T4. N = 6 biologically independent animals for T3 and N = 12 biologically

independent animals for T4 over 2 independent experiments. Student's unpaired two-tailed t-test. Data are presented as mean values ± standard deviation (SD). Male mice were analyzed. Source data are provided as a Source Data file (Source_Data).

Supplementary Figure 4

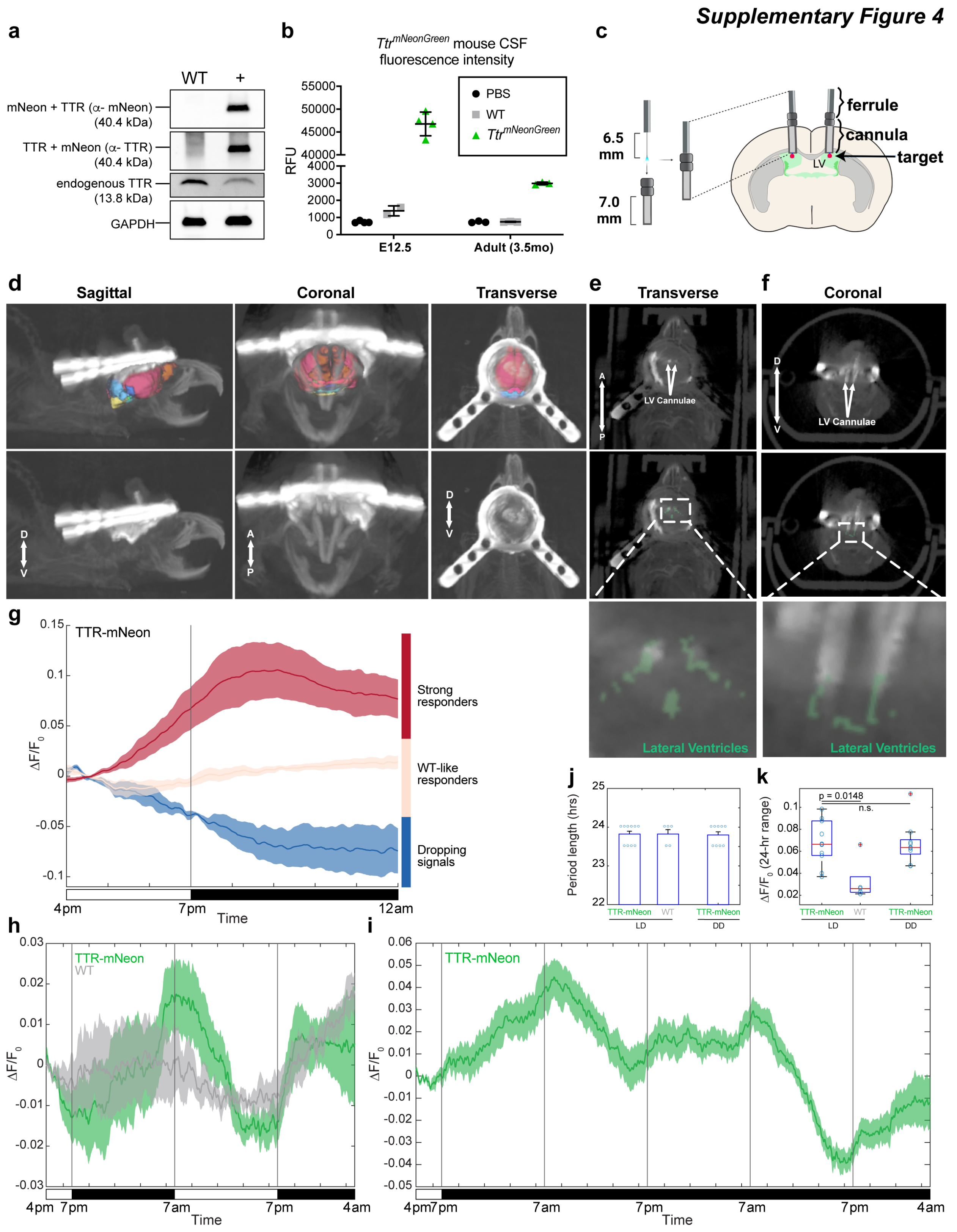

Supplementary Figure 4. Fiber photometry in *Ttr*^{mNeonGreen} mice reveals TTR cycles over multiple days that are light independent. (**a**) Immunoblotting of ChP from WT and *Ttr*^{mNeonGreen} mice probed for mNeonGreen, TTR, and GAPDH. (**b**) Fluorescent plate reader data of CSF from WT and *Ttr*^{mNeonGreen} mice compared to 1x PBS at E12.5 and Adult. N = 3-4 biologically independent animals at each age and genotype in one experiment. Lines indicate mean values ± standard deviation (SD). (**c**) Schematic of cannula and fiber placement in bilateral mouse LVs. 7mm-long cannulae were implanted at stereotactic coordinates ±1 lateral, -0.45 posterior, and -2 ventral to bregma. 6.5mm-long optical fibers were later placed in each cannula and secured with metabond. (**d**) Sagittal, coronal, and transverse perspectives on a 3D projected CT scan of a mouse with bilateral LV cannulae. A 3D model of the mouse brain is aligned and superimposed on each CT projection in the top panel. (**e**) A transverse CT demonstrates overlap between the projected lateral ventricles and bilateral guide cannulae. (**f**) A coronal CT demonstrates overlap between the projected lateral ventricles and bilateral guide cannulae. (**g**) Summary of *Ttr*^{mNeonGreen} recordings in **Figure 4c** subgrouped into three categories of responses (strong responders in red, wild-type-like in peach, dropping signals in blue, see **Figure 4d-e)**. Data are presented as mean values ± standard error of the mean (SEM). (**h**) Summary of all light-dark *Ttr*^{mNeonGreen} and wild-type recordings in the second 36 hours period starting just before the second lights off. N = 10 biologically independent *Ttr*^{mNeonGreen} animals across 4 independent experiments and N = 5 biologically independent wild-type animals across 2 experiments. Data are presented as mean values ± standard error of the mean (SEM). (**i**) Summary of all dark-dark *Ttr*^{mNeonGreen} recordings in the second 60 hours period starting just before the second lights off. N = 9 biologically independent *Ttr*^{mNeonGreen} animals across 3 independent experiments. Data are presented as mean values ± standard error of the mean (SEM). (**j**) RAIN analysis performed on the *Ttr*^{mNeonGreen} and wild-type light-dark recordings as well as the *Ttr*^{mNeonGreen} dark-dark recordings in **Supplementary**

Figures 4h-i shows that all signals have a consistent period just under 24 hours. Data are presented as mean values ± standard error of the mean (SEM). **(k)** Signal ranges over the first 24 hours of the plotted windows from the *Ttr*^{mNeonGreen} and wild-type light-dark recordings as well as the *Ttr*^{mNeonGreen} dark-dark recordings in **Supplementary Figures 4h-i** show that the *Ttr*^{mNeonGreen} animals demonstrate significantly larger signal ranges than the wild-types, and that the ranges in *Ttr*^{mNeonGreen} animals are consistent between light-dark and dark-dark conditions. Box plots show median value at the red central bar, with the bottom and top edges of the box indicating the 25th and 75th percentiles, respectively. The whiskers extend to the most extreme values not considered outliers, and outliers (more than 1.5 times the interquartile range away from median) are plotted individually and marked with a red '+' symbol. p = 0.0148 Student's unpaired two-tailed t-test, Bonferroni correction for 2 comparisons. Male mice were analyzed. Source data are provided as a Source Data file (Source_Data).

Supplementary Figure 5

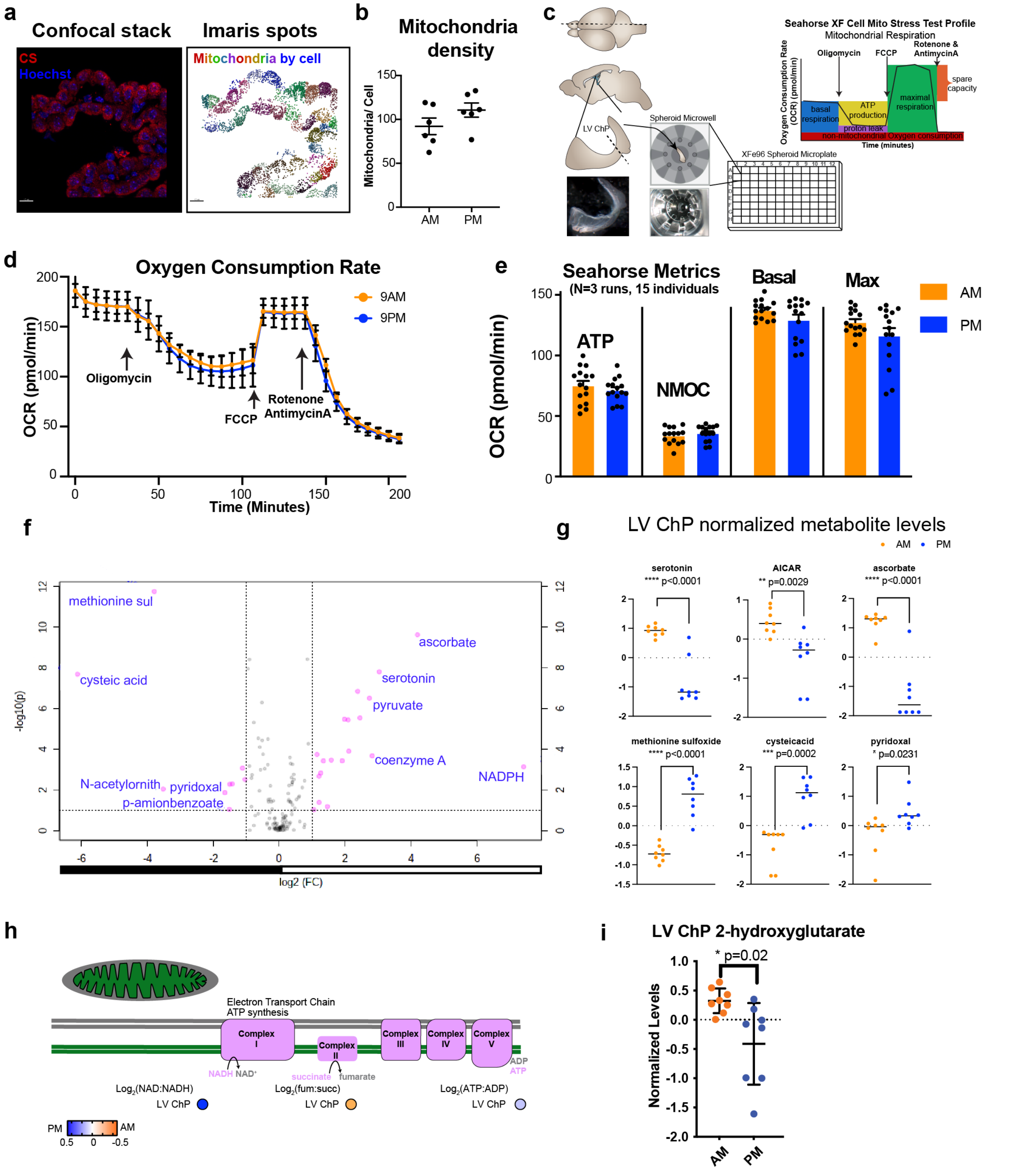

Supplementary Figure 5. *In vivo* ChP metabolic components and metabolites are diurnally regulated and *ex vivo* ChP metabolism is normalized between light and dark phases. (**a**) Immunostaining for citrate synthase (CS) in LV ChP alongside the Imaris (Bitplane) 'spots' quantification of mitochondria binned into individual cells. (**b**) Quantification of number of mitochondria (CS^+ spots) per LV ChP epithelial cell at 9 a.m. and 9 p.m. Error bars = S.E.M, each point is an average of mitochondria numbers from multiple cells. N = 7 biologically independent animals per time over 2 independent experiments. Data are presented as mean values ± standard deviation (SD). (**c**) Schematic and image of LV ChP dissection and explant for use in the XFe96 Spheroid microplate for Seahorse (Agilent) respiration analysis. Schematic of the Cell Mito Stress Test treatment used to assess *ex vivo* oxygen consumption of LV ChP. (**d**) Representative OCR (oxygen consumption rate) for a single run of the Cell Mito Stress Test on explanted LV ChP. One run is an average of 6 ChP from 3 biologically independent animals per time. Data are presented as mean values ± standard deviation (SD). (**e**) Meta-analysis of OCR (oxygen consumption rate) metrics from 3 technical replicates of the Cell Mito Stress Test in LV ChP. N = 15 ChP from 8 biologically independent animals per time over 3 independent experiments showed no difference in *ex vivo* ATP production, non-mitochondrial oxygen consumption (NMOC), basal respiration, or maximum respiration suggesting that ChP metabolism is influenced by external circadian cues. Two-tailed unpaired Student's t-test. Data are presented as mean values ± standard error of the mean (SEM). (**f**) Volcano plot of most substantial significantly differentially abundant metabolites in LV ChP at 9 a.m. (orange) and 9 p.m. (blue). (**g**) Relative values of differential metabolites in LV ChP at 9 a.m. (orange) and 9 p.m. (blue). N = 8 biologically independent animals per time. * $p < 0.05$; ** $p < 0.01$*** $p < 0.001$; **** $p < 0.0001$. Two-tailed unpaired Student's t-test. Data are presented as mean values ± standard deviation (SD). (**h**) Relative Log_2 ratios of electron transport chain (ETC) intermediates in LV ChP at 9 a.m. (orange) and 9 p.m. (blue). N = 8

biologically independent animals per time. (**i**) Relative values of 2-hydroxyglutarate in LV ChP at 9 a.m. (orange) and 9 p.m. (blue). N = 8 biologically independent animals per time. * $p < 0.05$. Two-tailed unpaired Student's t-test. Data are presented as mean values ± standard deviation (SD). Male mice were analyzed. Source data are provided as a Source Data file (Source_Data).

Supplementary Figure 6

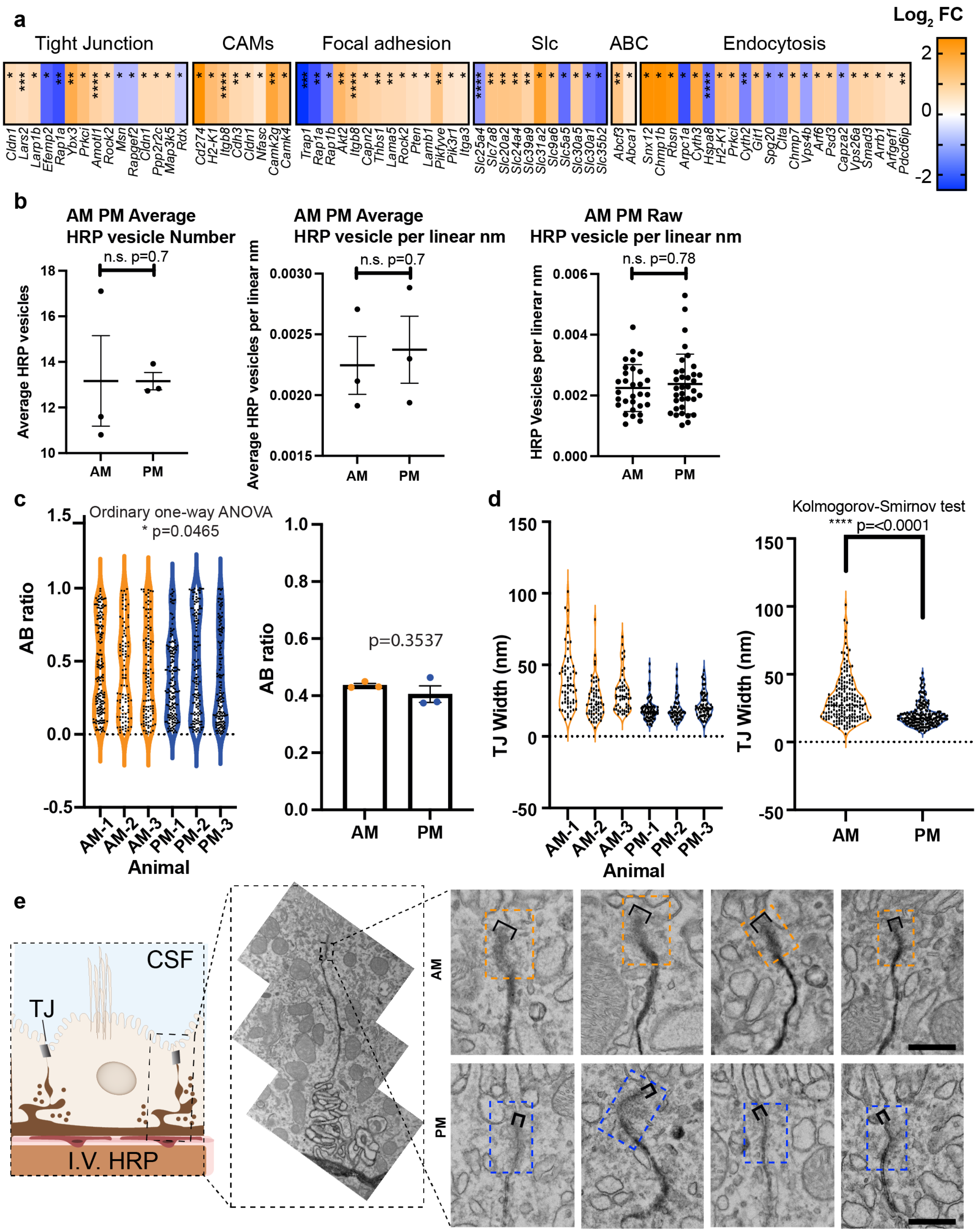

Supplementary Figure 6. ChP barrier components and permeability are diurnally regulated. (**a**) Heatmaps of Log_2FC of TRAP data related to barrier properties of the LV ChP at 9 a.m. (orange) and 9 p.m. (blue). (**b**) Quantification of HRP vesicles from mice at 9 a.m. and 9 p.m. Two-tailed unpaired Welch's t-test. Data are presented as mean values ± standard error of the mean (SEM). (**c**) Quantification of apical-basal ratio for all vesicles separated by each animal N = 3 and averaged within each animal. N = 3 biologically independent animals per time. Ordinary one-way ANOVA for first panel and two-tailed unpaired Student's t-test for second panel. Data are presented as mean values ± standard error of the mean (SEM). (**d**) Quantification of tight junction width for all measurements (10 per junction) separated by each animal N = 3 biologically independent animals per time and analyzed together for each timepoint. Kolmogorov-Smirnov test. Data are presented as violin plots with solid bar at the median and broken bars at quartiles. (**e**) Additional representative examples of TEM images of ChP epithelial cell tight junctions. Dotted boxes indicate analyzed region at the apico-lateral junctions between cells. Scale bars = 500 nm. Male mice were analyzed. Source data are provided as a Source Data file (Source_Data).
